# Supplementary material for: HIVprotI: an integrated web based platform for prediction and design of HIV proteins inhibitors
Source: J Cheminform. 2018 Mar 9;10:12. doi: 10.1186/s13321-018-0266-y (PMC5845081; doi:10.1186/s13321-018-0266-y)
Supplement: Supplementary file 2 — Additional file 2. Source code of HIVProtI web server. [file 13321_2018_266_MOESM2_ESM.zip › HIVprotI_Source-code/hivprotI/weka-3-6-6/doc/weka/core/Statistics.html]

Statistics


---


|  |  |  |
| --- | --- | --- |
| **Overview** \| **Package** \| **Class** \| **Tree** \| **Deprecated** \| **Index** \| **Help** \| **Weka's home** | |  |
| **PREV CLASS**   **NEXT CLASS** | **FRAMES**    **NO FRAMES**     **All Classes** |  |
| SUMMARY: NESTED \| FIELD \| CONSTR \| METHOD | DETAIL: FIELD \| CONSTR \| METHOD |  |


---


## weka.core Class Statistics

```
java.lang.Object
  weka.core.Statistics
```

**All Implemented Interfaces:**: RevisionHandler

---

``` public class Statistics extends java.lang.Object implements RevisionHandler ```

Class implementing some distributions, tests, etc. The code is mostly adapted from the CERN
Jet Java libraries:
Copyright 2001 University of Waikato
Copyright 1999 CERN - European Organization for Nuclear Research.
Permission to use, copy, modify, distribute and sell this software and its documentation for
any purpose is hereby granted without fee, provided that the above copyright notice appear
in all copies and that both that copyright notice and this permission notice appear in
supporting documentation.
CERN and the University of Waikato make no representations about the suitability of this
software for any purpose. It is provided "as is" without expressed or implied warranty.

**Version:**
:   $Revision: 5619 $

**Author:**
:   peter.gedeck@pharma.Novartis.com, wolfgang.hoschek@cern.ch, Eibe Frank (eibe@cs.waikato.ac.nz), Richard Kirkby (rkirkby@cs.waikato.ac.nz)

---

**Constructor Summary**

`Statistics()`


| **Method Summary** | |
| --- | --- |
| `static double` | `binomialStandardError(double p, int n)`             Computes standard error for observed values of a binomial random variable. |
| `static double` | `chiSquaredProbability(double x, double v)`             Returns chi-squared probability for given value and degrees of freedom. |
| `static double` | `errorFunction(double x)`             Returns the error function of the normal distribution. |
| `static double` | `errorFunctionComplemented(double a)`             Returns the complementary Error function of the normal distribution. |
| `static double` | `FProbability(double F, int df1, int df2)`             Computes probability of F-ratio. |
| `static double` | `gamma(double x)`             Returns the Gamma function of the argument. |
| `java.lang.String` | `getRevision()`             Returns the revision string. |
| `static double` | `incompleteBeta(double aa, double bb, double xx)`             Returns the Incomplete Beta Function evaluated from zero to xx. |
| `static double` | `incompleteBetaFraction1(double a, double b, double x)`             Continued fraction expansion #1 for incomplete beta integral. |
| `static double` | `incompleteBetaFraction2(double a, double b, double x)`             Continued fraction expansion #2 for incomplete beta integral. |
| `static double` | `incompleteGamma(double a, double x)`             Returns the Incomplete Gamma function. |
| `static double` | `incompleteGammaComplement(double a, double x)`             Returns the Complemented Incomplete Gamma function. |
| `static double` | `lnGamma(double x)`             Returns natural logarithm of gamma function. |
| `static void` | `main(java.lang.String[] ops)`             Main method for testing this class. |
| `static double` | `normalInverse(double y0)`             Returns the value, x, for which the area under the Normal (Gaussian) probability density function (integrated from minus infinity to x) is equal to the argument y (assumes mean is zero, variance is one). |
| `static double` | `normalProbability(double a)`             Returns the area under the Normal (Gaussian) probability density function, integrated from minus infinity to x (assumes mean is zero, variance is one). |
| `static double` | `p1evl(double x, double[] coef, int N)`             Evaluates the given polynomial of degree N at x. |

**Methods inherited from class java.lang.Object**

`equals, getClass, hashCode, notify, notifyAll, toString, wait, wait, wait`

**Constructor Detail**

### Statistics

```
public Statistics()
```


**Method Detail**

### binomialStandardError

```
public static double binomialStandardError(double p,
                                           int n)
```

:   Computes standard error for observed values of a binomial
    random variable.

    :   **Parameters:**: `p` - the probability of success: `n` - the size of the sample **Returns:**: the standard error

---


### chiSquaredProbability

```
public static double chiSquaredProbability(double x,
                                           double v)
```

:   Returns chi-squared probability for given value and degrees
    of freedom. (The probability that the chi-squared variate
    will be greater than x for the given degrees of freedom.)

    :   **Parameters:**: `x` - the value: `v` - the number of degrees of freedom **Returns:**: the chi-squared probability

---


### FProbability

```
public static double FProbability(double F,
                                  int df1,
                                  int df2)
```

:   Computes probability of F-ratio.

    :   **Parameters:**: `F` - the F-ratio: `df1` - the first number of degrees of freedom: `df2` - the second number of degrees of freedom **Returns:**: the probability of the F-ratio.

---


### normalProbability

```
public static double normalProbability(double a)
```

:   Returns the area under the Normal (Gaussian) probability density
    function, integrated from minus infinity to x
    (assumes mean is zero, variance is one).

    ```
                                x
                                 -
                       1        | |          2
      normal(x)  = ---------    |    exp( - t /2 ) dt
                   sqrt(2pi)  | |
                               -
                              -inf.

                 =  ( 1 + erf(z) ) / 2
                 =  erfc(z) / 2
    ```

    where z = x/sqrt(2).
    Computation is via the functions errorFunction and errorFunctionComplement.

    :   **Parameters:**: `a` - the z-value **Returns:**: the probability of the z value according to the normal pdf

---


### normalInverse

```
public static double normalInverse(double y0)
```

:   Returns the value, x, for which the area under the
    Normal (Gaussian) probability density function (integrated from
    minus infinity to x) is equal to the argument y
    (assumes mean is zero, variance is one).

    For small arguments 0 < y < exp(-2), the program computes
    z = sqrt( -2.0 \* log(y) ); then the approximation is
    x = z - log(z)/z - (1/z) P(1/z) / Q(1/z).
    There are two rational functions P/Q, one for 0 < y < exp(-32)
    and the other for y up to exp(-2).
    For larger arguments,
    w = y - 0.5, and x/sqrt(2pi) = w + w\*\*3 R(w\*\*2)/S(w\*\*2)).

    :   **Parameters:**: `y0` - the area under the normal pdf **Returns:**: the z-value

---


### lnGamma

```
public static double lnGamma(double x)
```

:   Returns natural logarithm of gamma function.

    :   **Parameters:**: `x` - the value **Returns:**: natural logarithm of gamma function

---


### errorFunction

```
public static double errorFunction(double x)
```

:   Returns the error function of the normal distribution.
    The integral is

    ```
                               x 
                                -
                     2         | |          2
       erf(x)  =  --------     |    exp( - t  ) dt.
                  sqrt(pi)   | |
                              -
                               0
    ```

    **Implementation:**
    For 0 <= |x| < 1, erf(x) = x \* P4(x\*\*2)/Q5(x\*\*2); otherwise
    erf(x) = 1 - erfc(x).

    Code adapted from the 
    Java 2D Graph Package 2.4,
    which in turn is a port from the
    Cephes 2.2
    Math Library (C).

    :   **Parameters:**: `a` - the argument to the function.

---


### errorFunctionComplemented

```
public static double errorFunctionComplemented(double a)
```

:   Returns the complementary Error function of the normal distribution.

    ```
      1 - erf(x) =

                               inf. 
                                 -
                      2         | |          2
       erfc(x)  =  --------     |    exp( - t  ) dt
                   sqrt(pi)   | |
                               -
                                x
    ```

    **Implementation:**
    For small x, erfc(x) = 1 - erf(x); otherwise rational
    approximations are computed.

    Code adapted from the 
    Java 2D Graph Package 2.4,
    which in turn is a port from the
    Cephes 2.2
    Math Library (C).

    :   **Parameters:**: `a` - the argument to the function.

---


### p1evl

```
public static double p1evl(double x,
                           double[] coef,
                           int N)
```

:   Evaluates the given polynomial of degree N at x.
    Evaluates polynomial when coefficient of N is 1.0.
    Otherwise same as polevl().

    ```
                         2          N
     y  =  C  + C x + C x  +...+ C x
            0    1     2          N

     Coefficients are stored in reverse order:

     coef[0] = C  , ..., coef[N] = C  .
                N                   0
    ```

    The function p1evl() assumes that coef[N] = 1.0 and is
    omitted from the array. Its calling arguments are
    otherwise the same as polevl().

    In the interest of speed, there are no checks for out of bounds arithmetic.

    :   **Parameters:**: `x` - argument to the polynomial.: `coef` - the coefficients of the polynomial.: `N` - the degree of the polynomial.

---


### incompleteGamma

```
public static double incompleteGamma(double a,
                                     double x)
```

:   Returns the Incomplete Gamma function.

    :   **Parameters:**: `a` - the parameter of the gamma distribution.: `x` - the integration end point.

---


### incompleteGammaComplement

```
public static double incompleteGammaComplement(double a,
                                               double x)
```

:   Returns the Complemented Incomplete Gamma function.

    :   **Parameters:**: `a` - the parameter of the gamma distribution.: `x` - the integration start point.

---


### gamma

```
public static double gamma(double x)
```

:   Returns the Gamma function of the argument.

---


### incompleteBeta

```
public static double incompleteBeta(double aa,
                                    double bb,
                                    double xx)
```

:   Returns the Incomplete Beta Function evaluated from zero to xx.

    :   **Parameters:**: `aa` - the alpha parameter of the beta distribution.: `bb` - the beta parameter of the beta distribution.: `xx` - the integration end point.

---


### incompleteBetaFraction1

```
public static double incompleteBetaFraction1(double a,
                                             double b,
                                             double x)
```

:   Continued fraction expansion #1 for incomplete beta integral.

---


### incompleteBetaFraction2

```
public static double incompleteBetaFraction2(double a,
                                             double b,
                                             double x)
```

:   Continued fraction expansion #2 for incomplete beta integral.

---


### getRevision

```
public java.lang.String getRevision()
```

:   Returns the revision string.

    :   **Specified by:**: `getRevision` in interface `RevisionHandler`
    :   **Returns:**: the revision

---


### main

```
public static void main(java.lang.String[] ops)
```

:   Main method for testing this class.


---


|  |  |  |
| --- | --- | --- |
| **Overview** \| **Package** \| **Class** \| **Tree** \| **Deprecated** \| **Index** \| **Help** \| **Weka's home** | |  |
| **PREV CLASS**   **NEXT CLASS** | **FRAMES**    **NO FRAMES**     **All Classes** |  |
| SUMMARY: NESTED \| FIELD \| CONSTR \| METHOD | DETAIL: FIELD \| CONSTR \| METHOD |  |


---
